# The Diverse Applications of Recombinant BCG-Based Vaccines to Target Infectious Diseases Other Than Tuberculosis: An Overview

**DOI:** 10.3389/fmicb.2021.757858

**Published:** 2021-10-21

**Authors:** Esma Mouhoub, Pilar Domenech, Momar Ndao, Michael B. Reed

**Affiliations:** ^1^The Department of Microbiology & Immunology, McGill University, Montreal, QC, Canada; ^2^The Infectious Diseases and Immunity in Global Health Program, Research Institute of the McGill University Health Centre, Montreal, QC, Canada; ^3^The McGill International TB Centre, McGill University, Montreal, QC, Canada; ^4^The Department of Medicine, McGill University, Montreal, QC, Canada; ^5^National Reference Centre for Parasitology, Research Institute of the McGill University Health Centre, Montreal, QC, Canada

**Keywords:** bacillus calmette-guerin, recombinant BCG, infectious diseases, vaccine development, bacterial vector

## Abstract

Live attenuated Bacillus Calmette-Guérin (BCG) is the world’s most widely used vaccine which is mainly administered for its protection against tuberculosis (TB), particularly in young children. However, since its initial use over 100years ago, it has also proven to offer a level of protection against various other pathogens, as a consequence of its non-specific immune enhancing effects. Thus, over the past few decades, recombinant BCG (rBCG) technology has been used as a vector to create rBCG vaccines expressing heterologous antigens that elicit immunity against a range of bacterial, viral, and parasitic diseases. Our goal with this mini-review is to provide an up-to-date survey of the various techniques, approaches, and applications of rBCG-based vaccines for targeting infectious diseases other than TB.

## Introduction

BCG is an attenuated form of the *Mycobacterium bovis* (*M. bovis)* bacteria, a close relative of *Mycobacterium tuberculosis* (*M. tb*), and is currently used to vaccinate infants against TB meningitis and miliary disease (Mahairas et al., 1996; Zimmermann et al., 2019). According to the recently updated BCG Atlas, BCG is the most universally given vaccine across the globe with over 160 million infants receiving the vaccine annually (Zimmermann et al., 2019), although long-term efficacy may vary globally (Angelidou et al., 2020). The benefits of BCG vaccination include its heat stability, easy mass production, low cost, and safe neonatal use, it is unaffected by maternal antibodies, and it is associated with induction of non-specific heterologous immunomodulatory effects (Kilpeläinen et al., 2019; Zimmermann et al., 2019). Indeed, BCG immunization at birth induces cross-protection for a number of infectious pathogens including *M. tb, Candida albicans, Staphylococcus aureus*, respiratory syncytial virus (RSV), influenza A virus, and herpes simplex virus type 2, due to its non-specific immunomodulatory, or adjuvant-like, effects (Starr et al., 1976; Spencer and Ganguly, 1977; Van’t Wout et al., 1992; Kleinnijenhuis et al., 2012; Nemes et al., 2018; Covián et al., 2019; O’Neill and Netea, 2020). While an in-depth discussion of the various immune-related mechanisms associated with BCG is beyond the scope of the present article, in recent years, it has been demonstrated that BCG is able to trigger the epigenetic reprogramming of innate immune cells (“trained immunity”) that may function as APC (antigen-presenting cells), as well as augmenting T-cell immunity by stimulating IFN-γ production and subsequent Th1 cellular responses (Kleinnijenhuis et al., 2012, 2015; Zimmermann et al., 2019).

The BCG bacterium now exists globally as a range of genetically distinct substrains that lack varying genomic regions relative to *M. tb* and *M. bovis*. The common deletion of RD1 (Region of Difference 1) that is present in all BCG strains is a major cause of attenuation (Conrad et al., 2017; Tiwari et al., 2019). The latter encodes a vital part of the type VII secretion system of *M. tb*, known as ESX-1 (Majlessi et al., 2005). Although BCG diversification has led to heterogeneous substrains, to date, no significant difference in efficacy has been reported in humans among the various substrains (Milstien and Gibson, 1990; Behr and Small, 1999). In animal studies, the immunogenicity of BCG appears to vary in a strain, dose, and delivery-dependent manner. In addition, comparative analyses in mice have reported differences in efficacy and virulence across 13 diverse BCG substrains (Zhang et al., 2016). Low doses of BCG elicit an almost exclusively Th1 response, while higher doses elicit a mixed Th1/Th2 response (Jiao et al., 2003). In addition, although BCG is delivered to infants intradermally, alternative delivery methods may enhance specific immune responses. For example, intranasal administration of BCG is a method of potentially inducing systemic and long-lasting secretory immune responses (Langermann et al., 1994; Angelidou et al., 2020). Therefore in the future design of BCG-based vaccines, the availability of BCG substrains already in use in the target population, specific environmental factors, and administration techniques may all be important variables to account for (Castillo-Rodal et al., 2006).

BCG’s ability to induce a robust Th1 response and non-specific immunomodulatory effects make it a successful “live-adjuvant” for combining with a variety of heterologous antigens. In the past, the direct co-administration of BCG mixed with an antigen of interest has been examined for protection against various diseases. For example, Pearce et al. have used paramyosin, an antigen derived from *Schistosoma mansoni*, for intradermal co-administration with BCG in mice. Partial protection of 26–33% against *S. mansoni* challenge occurred when BCG was co-administered with only 4–40μg of the paramyosin antigen, while 2mg of the antigen alone was required to obtain the same level of protection. Protection occurred through the induction of a dominant Th1 response (Pearce et al., 1988; Sher et al., 1991). In a related study in sheep, co-administration of BCG and paramyosin leads to enhanced immunogenicity and up to 48% worm reduction compared to BCG alone (Taylor et al., 1998). Furthermore, the use of BCG as an adjuvant alongside various autoclaved *Leishmania* has proven to be a promising combination and is widely used in *Leishmania* vaccine (Dube et al., 1998; Misra et al., 2001; Nahrevanian et al., 2013). More recently, the progression of recombinant DNA technology has led to the utilization of BCG as a vaccine platform *via* genetic engineering approaches. Originally described in 1991 in a landmark paper by Stover et al., foreign genes of interest can now routinely be introduced into BCG through the use of *E.coli-mycobacterium* shuttle vectors, such that the encoded proteins are expressed extracellularly, intracellularly, or remain bound to the BCG cell surface (Stover et al., 1991; Zheng et al., 2015). In an attempt to maintain a stable level of expression of foreign antigens in BCG, the mycobacterial *hsp60* promoter is most commonly included in plasmid expression vectors that are capable of integrating at the non-essential mycobacteriophage L5 attachment site (attB; Stover et al., 1991; Zheng et al., 2015). Lastly, optimization of foreign gene sequences based on the codon-usage preferences observed in Mycobacteria may also prove beneficial to maximize the level of heterologous gene expression (Varaldo et al., 2004).

### rBCG Applications and Approaches

#### Anti-viral Vaccine Strategies

rBCG vaccines have been designed using a wide range of viral, parasitic, and bacterial antigens including toxins. rBCG is a strong vaccine platform for viral diseases due to its ability to induce a strong Th1 response, which is ideal for clearing intracellular pathogens (Rosenthal and Zimmerman, 2006; Abarca et al., 2020). In HIV research, rBCG vaccines using HIV-specific antigens have been created to elicit virus-specific T-cell responses, generally using a prime-boost regimen. Priming with rBCG expressing HIV antigens and boosting with different vectors expressing HIV antigens appears to be favorable in HIV vaccine studies partially due to longer lasting immune effects – such as longer highly specific CTL responses – when compared to vaccinating with rBCG alone (Promkhatkaew et al., 2009a,b). For example, Kilpeläinen et al. primed BALB/c mice with rBCG expressing conserved HIV-1 mosaic immunogens and boosted them with simian adenovirus containing additional HIV-1 mosaic immunogens (Kilpeläinen et al., 2019). Inoculation was safe and led to the induction of HIV-1 specific IFN-γ producing T-cell responses and cytokine secretion, which were both significantly higher in the primed mice (Kilpeläinen et al., 2019). In addition, multiple rBCG vaccine constructs have been developed for testing against simian immunodeficiency (SIV) as a model for HIV disease. Many HIV researchers have used the gag polyprotein, which mediates vital viral assembly events, as an antigen due to its induction of cellular immunity (Jalalirad and Laughrea, 2010; Martins et al., 2014). Ami et al vaccinated cynomolgus macaques with rBCG and a replication-deficient vaccinia virus boost, both expressing full-length SIV gag (Ami et al., 2005). Upon challenge with a recombinant pathogenic simian-human immunodeficiency virus (SHIV KS661c), viral control, and effective long-lasting immunity through antigen-specific IFN-γ production was only observed in the macaques primed with rBCG, but not the control groups (Ami et al., 2005). More recently, Kato et al primed cynomolgus monkeys with rBCG expressing SIV *gag* followed by a boost with SIV *env* or SIV *pol* expressing Vaccinia and a second boost with SIV *env*-expressing Sendai virus (Kato et al., 2020). Upon repeated challenge with SIVmac251, a pathogenic antibody-resistant (tier 2) SIV strain previously demonstrated to mimic HIV-1, plasma viremia was significantly lower, and in some cases undetectable (Kato et al., 2020). Moreover, high levels of potent CD8+ T cells were observed in vaccinated monkeys, but not the unvaccinated controls (Kato et al., 2020). Interestingly, the rBCG strain used here was urease-deficient, which seemed to increase immunogenicity against both BCG and SIV (Kato et al., 2020). Urease is involved in neutralization of the BCG-containing phagosome and urease-deficient BCG has been shown to be useful in T-cell activation by more efficiently producing memory T cells in C57BL/6 mice, and potentially increasing presentation of BCG-derived antigens for CTL induction (Mukai et al., 2008; Nieuwenhuizen et al., 2017; Kato et al., 2020). Although substantial research has been focused on using rBCG for HIV, a number of other viral vaccine candidates are being created. A recent phase I clinical trial for an rBCG RSV vaccine conducted by Abarca et al demonstrated that participants could safely tolerate a rBCG-RSV vaccine containing the N protein of RSV (Abarca et al., 2020). The participants had serum IgG-antibodies specific to the RSV N protein, along with an increased cellular Th1 response specific to both the N protein and mycobacterial PPD (purified protein derivative; Abarca et al., 2020). Additional examples of viral candidate vaccines produced in the rBCG background include hepatitis C Virus (HCV; Wei et al., 2008) and human metapneumovirus (hMPV; Palavecino et al., 2014; Table 1).

**Table 1:** rBCG vaccine candidates and their immune responses against various viral, parasitic, or bacterial challenges.

	Infection	BCG Strain	Gene(s)/Protein(s) expressed	Additional Antigen Details	Animal model	Type(s) of immune response noted	References
Viral	Human immunodeficiency virus 1 (HIV-1)	Danish and Connaught	HIVconsv1&2 immunogens along with ChAdOx1.tHIVconsv5&6 (conserved HIV-1 mosaic immunogens)	Mtb19-kDa lipoprotein signal sequence	BALB/c mice	Cellular: Increased induction of IFN-γ and TNF-α, CD4+ and CD8+ CTLs.	Kilpeläinen et al., 2019
	Simian immunodeficiency virus (SIV)	Tokyo	SIVgag polyprotein	α antigen, extracellular secretion of fusion protein	Male cynomolgus macaques *(Macaca fascicularis)*	Prime-boost inoculation of rBCG-SIVgag followed by rDIsSIVgag lead to cellular responses through increased IFN-γ and immunity against SHIV challenge for one year.	Ami et al., 2005
		Tokyo	SIV proteins: Gag, Env gp120 and a fusion protein of Rev., Tat and Nef (RTN) proteins	Urease-deficient BCG expressing SIV genes	Male cynomolgus macaques	Cellular: little to no plasma viremia detected, along with high levels of potent CD8+ T cells were observed, and partial or full protection from challenge.	Kato et al., 2020
	Hepatitis C virus (HCV)	Tokyo	CtEm, a multi-epitope antigen composed of HCV structural and non-structural epitopes	Signal peptide α-ss antigen derived from H37Rv Mtb, secreted CtEm protein	HLA-A2.1 transgenic mice	Th1 dominant cellular response and induction of specific anti-HCV antibodies. Protection against recombinant vaccinia virus (rVV-HCV-CNS) was observed *in vivo*.	Wei et al., 2008
	Human metapneumovirus (hMPV)	Danish	M2-1 (participates in viral transcriptional regulation) and hMPV-P (phosphoprotein)		BALB/c mice	Th1 dominant response, induction of hMPV-specific T cells producing IFN-γ and IL-2, immunized mice were protected against disease symptoms and viral replication in the lungs.	Palavecino et al., 2014
	Respiratory syncytial virus (RSV)	Danish	RSV nucleoprotein (N)		Phase I clinical trial: 24 healthy males aged 19–44	Serum IgG-antibodies directed against Mycobacterium and RSV N protein, induced after vaccination and neutralized RSV *in vitro*. Increased IFN-γ and IL-2 also observed.	Bueno et al., 2008; Abarca et al., 2020
	Rotavirus	Tokyo and Pasteur	VP6 (immunogenic intermediate-layer capsid protein)	Mtb19-kDa lipoprotein signal sequence, VP6 linked to BCG cell membrane	BALB/c mice	Up to 66% reduction in fecal viral shedding compared to controls upon rotavirus challenge. No anti-rotavirus antibody was detected, meaning antigen-specific CD4^+^ or CD8^+^ T cells are most likely mediators of protection from viral infection.	Dennehy et al., 2007
Parasitic	*Plasmodium falciparum*	Glaxo	CSp (circumsporozoite protein)		BALB/c mice	CS-specific antibodies and IFN-γ producing memory cells, along with increased activation of APCs for priming adaptive immunity.	Arama et al., 2012
	*Toxoplasma gondii*	Pasteur	TgCyP (cyclophilin)		BALB/c mice	Dominant Th1 response through high levels of IFN-γ, IL-2 and IL-12, especially after oral administration. IV administration led to increased survival time and survival rate, along with high IgG specific antibody production.	Yu et al., 2013
	*Trypanosoma cruzi*	Pasteur	NT-TS (N and C terminal of trans-sialidase) and CZf (cruzipain enzyme) fragments		BALB/c mice	Increased level of protection and decreased level of parasitemia after challenge. Th1/Th17 responses observed through induction of IFN-γ, IL-17 and CD107 expression.	Bontempi et al., 2020
	*Eimeria maxima*	N/A	AMA1 (apical membrane antigen1 of *E. maxima*)	AMA1 present in the cellular lysate of rBCG.	One-day-old specific pathogen-free chickens	Intranasal and subcutaneous immunization lead to reduced disease symptoms. Intranasal immunization led to serum antibody, CD4+ and CD8+ T cells, IL-1β, IFN-γ, IL-15, and IL-10 induction.	Li et al., 2013
	*Schistosoma mansoni*	Pasteur	Sm14 (fatty-acid binding protein of *S. mansoni*)	Complete *Mycobacterium fortuitum* β-lactamase protein sequence (Blam) fused to the Sm14 sequence.	BALB/c or Swiss mice	Th1 response through increased levels of IFN- γ, and 48% reduction in worm burden compared to nonvaccinated controls.	Varaldo et al., 2004
Bacterial Toxins/Antigens	Shiga toxin-producing *Escherichia coli*(STEC)	Tokyo	Stx2 B subunit (nontoxic shiga toxin)		BALB/c mice	Immunization led to humoral responses (protective serum IgG and mucosal IgA) and longer survival upon oral challenge.	Fujii et al., 2012
	*Leptospira interrogans*	Pasteur	Four constructs composed of *lipL32, lemA* and *ligANI* genes (conserved, exposed L*eptospira* antigens)		Golden Syrian hamsters	Cellular Th1 immunity and 100% protection against leptospirosis.	Dorneles et al., 2020
	*Bordetella pertussis*	Moreau	S1 subunit of PT-9K/129G (genetically detoxified S1 pertussis toxin genes)		Swiss mice	Th1 dominant immune response (IFN- γ and TNF-α induction) and high levels of protection against intracerebral challenge.	Nascimento et al., 2009; Kanno et al., 2019
	*Borrelia burgdorferi*	Pasteur	Outer surface protein A (OspA)	Mtb19-kDa lipoprotein signal sequence, OspA expressed as a membrane-associated lipoprotein	Swiss, BALB/c, or C3H/HeJ mice	100-1,000-fold higher protective antibody response when expressed as a membrane-associated lipoprotein when compared to cytoplasmic or secreted protein expression.	Stover et al., 1993

#### Anti-parasitic Vaccines

TB is a leading cause of death in sub-Saharan Africa, where individuals are commonly co-infected with parasitic diseases (Brooker et al., 2006). Comorbidity between parasitic and bacterial diseases creates a contrasting scenario since helminths tend to induce strong Th2 responses, while TB induces a Th1-biased response (Cozmei et al., 2007; Cadmus et al., 2020). This situation creates a potential challenge for the development of rBCG protecting against both infant TB and parasitic diseases. Malaria, a life-threatening disease caused by the apicomplexan parasite *Plasmodium falciparum*, has been targeted by rBCG vaccines. For example, a study conducted by Arama et al immunized BALB/c mice with rBCG expressing the *P. falciparum* circumsporozoite protein (CSp) and observed an upregulation of MHCII activation, along with CSp-specific IFN-γ producing memory cells and antibodies (Arama et al., 2012). Mice inoculated with a homologous BCG-CSp prime-boost regimen had the most robust Th1 response, characterized by higher levels of IgG2a antibodies compared to the homologous CSp/CSp recombinant protein group (Arama et al., 2012). However, this vaccine’s efficacy against *P. falciparum* challenge has yet to be documented in mice (Arama et al., 2012; Minkah et al., 2018). As previously mentioned, vaccines have been developed for *Schistosoma* by co-administrating BCG with a *Schistosoma* antigen, such as paramyosin and Sm23 (Pearce et al., 1988; Taylor et al., 1998). More recently, rBCG expressing the Sm14 antigen (a fatty-acid binding protein) from *S. mansoni* has been created and proven to induce a predominantly Th1-type-response in BALB/c or Swiss mice (Varaldo et al., 2004). Furthermore, one or two doses of the rBCG vaccine conferred a 48% reduction in worm burden upon challenge, which was comparable to three doses of the purified rSm14 antigen (Varaldo et al., 2004). Although rBCG induces a predominantly Th1-type-response, Th2 responses may still be observed with rBCG parasitic vaccines. In the case of *Toxoplasma gondii*, an opportunistic protozoan parasite causing severe toxoplasmosis in humans and livestock, following vaccination with BCG expressing *T. gondii* cyclophilin (TgCyP) – a nitric oxide inducing protein vital for the *T. gondii* life cycle (Ibrahim et al., 2009) – both Th1 and Th2 responses were observed in BALB/c mice prior to pathogenic *T. gondii* challenge (Yu et al., 2013). Although the Th1 response was much higher in the rBCG vaccinated groups, Th2 responses were equivalent in control (PBS or BCG) and rBCG groups (Yu et al., 2013). Therefore, rBCG vaccination may be able to induce an enhanced Th1 response without reducing or interfering with the critical Th2 responses naturally induced against parasitic infections (Yu et al., 2013). However, a limited protection of only 17% was reached in these vaccinated mice when challenged with *T. gondii* (Yu et al., 2013). Targeting parasitic proteins through rBCG have also been analyzed for *Trypanosoma cruzi* and *Eimeria maxima* (Li et al., 2013; Bontempi et al., 2020; Table 1).

#### Anti-bacterial and Anti-toxin Strategies

Several bacterial antigens have also been expressed in BCG in an attempt to induce immunity. A recent study by Dorneles et al used various combinations of Leptospira antigens to target the zoonotic bacterial disease leptospirosis (Dorneles et al., 2020). Upon subcutaneous inoculation of hamsters with four rBCG constructs, each encoding a distinct portion of a recombinant chimera made of various leptospiral antigens, all constructs provided full protection against leptospirosis challenge through the induction of cellular immune responses, in contrast to the BCG controls which had no protection (Dorneles et al., 2020). However, rBCG anti-bacterial vaccines have also been shown to elicit strong humoral responses. For example, in one of the earliest descriptions using BCG as a vaccine platform, an rBCG vaccine expressing the OspA outer surface protein antigen of *Borrelia burgdorferi* was shown to generate high titer antibody responses across a diverse set of heterogeneous mouse strains (Stover et al., 1993). Furthermore, this protective antibody response was observed to be 100-1,000-fold higher when OspA was expressed as a membrane-associated lipoprotein, compared to cytoplasmic or secreted forms of the same protein (Stover et al., 1993). Additional bacterial diseases whose toxins or antigens have been expressed within rBCG strains include Shiga toxin-producing *E. coli* (STEC) and *Bordetella pertussis* (Nascimento et al., 2009; Fujii et al., 2012). Both vaccines induced high levels of protection against bacterial challenge through differing immune responses. In the case of the rBCG STEC vaccine, protection against STEC oral challenge was observed through humoral immunity, specifically the induction of protective serum IgG and mucosal IgA responses (Fujii et al., 2012). However for the rBCG-Pertussis vaccine, a predominantly cellular Th1 response involving IFN-γ and TNF-α release was associated with protection against lethal intracerebral challenge of *B. pertussis* in neonate mice (Nascimento et al., 2009). Therefore, rBCG can elicit lasting humoral and cellular immunity for various foreign bacterial antigens (Zheng et al., 2015).

## Discussion

BCG is an extremely advantageous platform for vaccine development due to its long-history of being safe to use (it is approved for use in many countries worldwide), its stability, and ease of distribution, and it is a live-vaccine vehicle that provides sustained cellular immune responses. It also serves as an effective adjuvant by enhancing immunogenicity above that seen with just the antigen alone (Varaldo et al., 2004; Zheng et al., 2015) and is capable of “off-target” immune enhancing effects including trained immunity (Vierboom et al., 2021). When employing rBCG, surface expression or secretion of the protein of interest appears to be critical, partially due to increases in antigen presentation and improved cross-priming, both leading to increased cellular immunity (Deres et al., 1989; Reitermann et al., 1989; Grode et al., 2005; Sali et al., 2010). Therefore, as noted above and in Table 1, signal sequences, such as those derived from Ag85B and the 19kDa antigen, are commonly fused to the foreign genes of interest (Garbe et al., 1993; Gomez et al., 2000; Sixsmith et al., 2014; Oliveira et al., 2017). In many instances, it may be ideal to choose a signal sequence which utilizes the general secretory pathway (Bendtsen et al., 2005). The latter may be favorable since it transports proteins in an unfolded state, thus limiting the creation of non-functional misfolded and aggregated proteins and does not require the action of chaperone proteins or accessory molecules (Feltcher et al., 2010). Bacterial lipoprotein signal sequences, such as that associated with the 19kDa lipoprotein (LpqH), have also been used in rBCG research due to their strongly immunogenic properties (Stover et al., 1993; Dennehy et al., 2007). For example, an anti-rotavirus vaccine expressing VP6 only induced significant cellular immunity-based protection when linked to the 19kDa lipoprotein signal sequence that allowed transport of VP6 to the BCG outer membrane (Dennehy et al., 2007).

One potential limitation in the use of rBCG technology exists in the use of *E. coli-mycobacterium* shuttle vectors to transfer foreign genes of interest into BCG. These plasmids commonly contain an antibiotic resistance gene as a selectable marker. However, vaccines containing antibiotic genes are largely unsuitable for human use, predominantly due to the potential risk of horizontal transfer of antibiotic resistance to nearby microbial populations (Borsuk et al., 2007; Mignon et al., 2015). Therefore, methods, such as Cre-lox recombination, have been explored to subsequently remove antibiotic resistance markers following transformation and selection. Cre-lox recombination systems are very efficient for multiple gene replacements but to the best of our knowledge have yet to be applied to rBCG development, although they have been used in other Mycobacteria (Lambert et al., 2007; Murphy et al., 2015; Hurst-Hess et al., 2017). Alternate selection methods, such as auxotrophic complementation, allow *in vivo* selection in the absence of antibiotics through the use of a BCG strain that, for example, is auxotrophic for leucine (*leuD* knockout) in conjunction with a plasmid vector containing *leuD* that is then used to introduce the foreign antigen of interest (Borsuk et al., 2007). The creation of an unmarked rBCG vaccine in this manner has been described by Nascimento et al. (2009). In this particular study, a rBCG-pertussis vaccine, which elicited protection against *B. pertussis* challenge in mice, was generated through auxotrophic complementation based on lysine rather than leucine (Nascimento et al., 2009).

In summary, BCG has been shown to provide protection against a large range of infections beyond TB, including both bacterial and viral respiratory infections (Fine, 1995; Zheng et al., 2015). Even in the context of the current COVID-19 pandemic, a correlation has been suggested between COVID-19 case fatality rates and national rates of BCG vaccination (Rivas et al., 2021). Although it remains to be proven, protection against SARS-CoV-2 could potentially be associated with BCG’s trained immunity and non-specific Th1 responses, in line with its ability to reduce other respiratory tract infections in children (Rivas et al., 2021). In theory, any anti-SARS-CoV-2 activity displayed by BCG could potentially be enhanced by creating a novel rBCG-based vaccine candidate that expresses one or more protein components of the SARS-CoV-2 virus (Krammer, 2020) and our group is currently exploring this line of inquiry.

## Conclusion

The use of BCG for a variety of vaccination purposes – both specific and non-specific – has opened the door for renewed enthusiasm to further explore the use of rBCG vaccine technology in future vaccine development and immunotherapy.

## Author Contributions

EM performed the literature searches and wrote the manuscript. MR, PD, and MN contributed to the writing and editing. All authors contributed to the article and approved the submitted version.

## Funding

This manuscript was supported by funding obtained from the McGill Interdisciplinary Initiative in Infection and Immunity (MI4).

## Conflict of Interest

The authors declare that the research was conducted in the absence of any commercial or financial relationships that could be construed as a potential conflict of interest.

## Publisher’s Note

All claims expressed in this article are solely those of the authors and do not necessarily represent those of their affiliated organizations, or those of the publisher, the editors and the reviewers. Any product that may be evaluated in this article, or claim that may be made by its manufacturer, is not guaranteed or endorsed by the publisher.
